# Uptight responses between clenching and forearm raising with factors of visual feedback and maintenance effort in healthy young women: An experimental study on factorial design

**DOI:** 10.1186/s12903-023-02767-9

**Published:** 2023-02-14

**Authors:** Lijuan Zhou, Baoyong Li, Xianyu Zheng, Shaoxiong Guo, Yuan Zhang, Changsheng Chen, Kelun Wang, Meiqing Wang

**Affiliations:** 1 Shanghai Key Laboratory of Craniomaxillofacial Development and Diseases, Fudan University, Shanghai, China; 2 Department of Orthodontics I, Hefei Stomatological Hospital, Clinical College of Stomatology, Anhui Medical University, Hefei, 230001 Anhui China; 3 Stomatologic Hospital & College, Anhui Medical University, Key Lab. of Oral Diseases Research of Anhui Province, Hefei, 230032 China; 4 Department of Oral Anatomy and Physiology, School of Stomatology, The Fourth Military Medical University, Xi’an, 710032 Shaanxi China; 5 Department of Health Statistics, The Fourth Military Medical University, Xi’an, 710032 Shaanxi China; 6 Center for Sensory-Motor Interaction (SMI), Department of Health Science and Technology, Faculty of Medicine, Aalborg University, Fredrik BajersVej 7 D3, 9220 Aalborg, Denmark

**Keywords:** Psychological responses, Biting force, Jaw-closing muscle, Biceps brachii muscle, Electromyography

## Abstract

**Background:**

To achieve different central preset force levels requires various fine-tuning efforts and may elicit different uptight responses. The mandibular lever system has a distinct regularity in the fine-tuning function of the upper limbs. The purpose of the present study was to detect whether the uptight responses elicited from motivating clenching differ from those induced by motivating forearm raising at different force levels.

**Methods:**

Twenty-five healthy females were enrolled in this study. The target was low, medium, and maximum force levels with or without visual feedback and/or maintenance effort. Surface electromyographic (SEMG) activity was recorded from the bilateral anterior temporalis and masseter or left biceps brachii muscle (BicL), and the T-Scan III System synchronously recorded the sensitive force values. The uptight responses and task difficulties were recorded for occlusal and left forearm lifting tasks using a unique visual analogue scale.

**Results:**

The highest uptight response value was achieved at a low clenching force level with visual feedback requiring no maintenance effort but at a maximum forearm-raising force level with visual feedback and maintenance effort. The SEMG activities of both jaw-closing muscles and BicL were associated with the central preset force level (*P* < 0.001). However, the maintenance effort only increased the jaw-closing muscles’ SEMG activity at the maximal force level (*P* < 0.001).

**Conclusions:**

Clenching at the central preset lower force level with visual feedback is prone to elicit a higher degree of uptight response. The constant need for a low-intensity bite can have a negative effect on an individual's mood.

**Supplementary Information:**

The online version contains supplementary material available at 10.1186/s12903-023-02767-9.

## Background

When humans perform a difficult task, it elicits a physical tightening or tensing response, which is most likely due to incessantly peripheral modifications of the central motivating behaviour. Chewing cycles exhibit a complex pattern through cerebrocentric motivation and incessant peripheral feedback [1]. The involvement of peripheral modification of occlusion on chewing function is supported by the jaw-closing velocity being lower than when open during chewing [2, 3]. Moreover, at the initiation of the occlusal phase of a chewing cycle, there can be a short silent period in the jaw muscles [4]. The modification of occlusion on motor function can be presented as some patterns of orofacial development and functional behaviours. During development, central and peripheral mastication leads to intellectual mastication, which generates accommodative jaw, tongue, and lip movements [5]. In twin population observations, a larger jaw volume was found on the chewing side, suggesting that functional factors were more obvious than genetic factors [6]. Therefore, an asymmetrical condylar head and body length are observed in patients with a unilateral posterior crossbite [7]. However, in adulthood, this peripheral modification to central motor activity may lead to functional disorders, such as chewing-side preference and tiredness or even fatigue pain of the orofacial region. The former should be a passive choice of rhythmic movement to prevent potential discomfort or harm. The latter can be observed during persistent clenching or grinding [8]. Individuals with temporomandibular disorders (TMDs), abnormal chewing, malocclusion, and oral parafunctional habits such as bruxism have a high prevalence of psychological distress [9]. Hanna et al. found that higher impairments were observed in physical pain, psychological disability, and psychological discomfort in TMDs patients; further, oral health-related quality of life (OHRQoL) was negatively affected among TMDs patients [10]. People with occlusal disharmony may experience chronic orofacial pain and often suffer psychological distress, sleep disturbance and poor quality of life [11].

Furthermore, the problematic modification may lead to an individual's psychological changes, such as uptight responses, which means a response stemming from a state of emotional distress. However, such uptight response-related clenching discomfort remains unaddressed. It is worth clarifying what type of centrally motivated and peripherally modified clenching is prone to inducing uptight responses.

The T-Scan III system is an occlusion pressure recorder that can display task force information on a screen. Surface electromyography (SEMG) activity is positively linked to muscular contraction strength. It provides the sum of data on the electrical contributions of the active motor units [12], thus reflecting the muscle activation properties and the central control strategies [13]. In the current study, we conducted a cerebrocentric motivating clenching test at mentally preset force levels. We used the T-Scan System as a force indicator, with/without visual feedback modification and with/without maintenance effort. The SEMG activity of the bilateral anterior temporalis (TA) and masseter (MM) muscles was recorded. Limb movements, which are stimulated by muscle proprioceptors and exteroceptors, also depend on the afferent input relayed to sensory areas of the brain [14]. Sensory input from periodontal mechanoreceptors, a structural mechanism of force execution or fine-tuning, is different from that of other body parts, such as the hand [15]. Similar peripheral feedback mechanisms, such as visual feedback on motor function, have been studied [16], but studies on the orofacial role are scarce. It is interesting to consider limb action as a control task. The current investigation aimed to detect whether motivated clenching at different force levels with or without visual feedback and/or maintenance effort elicited different degrees of uptight responses and whether the clenching-induced uptight responses displayed a different regularity from those induced by forearm raising.

## Methods

### Subjects

Twenty-five healthy right-handed females (25.2 ± 4.5 years old; body weight, 51.7 ± 6.0 kg; body height, 162.7 ± 3.5 mm; and length of the left upper limb, 51.9 ± 1.9 mm) were recruited from the Fourth Military Medical University (FMMU). All subjects had 28–32 teeth that arranged well with the Class I molar relationship and optimal 2 to 5 mm overbite and overjet. The exclusion criteria included known signs, symptoms, or history of temporomandibular disorders, craniocervical disorders [17], previous craniofacial trauma, bruxism history, known periodontal problems, history of tooth restorations or orthodontic treatment or orthognathic surgery, gum-chewing habit (over 30 min a day) [18], or disease history of the upper limbs. All volunteers signed informed consent, and the FMMU Institutional Review Board Committee approved the procedures. The study was conducted following the ethical standards in the 1964 Declaration of Helsinki.

### Postural preparation

Each volunteer was seated comfortably in a chair, with back supported, feet flat on the floor, eyes fixed on a mark approximately two metres in front of her at eye level, head upright with the Frankfort horizontal plane parallel to the ground. For clenching tasks, the participants were asked to clench from the resting position to the intercuspal position (ICP) at the required force level indicated on the screen. For the biceps brachii muscles measurement, the subjects were seated upright with their elbows flexed at 135°, which was verified using a goniometer. They were instructed to lift from the resting position upwards to the required force level.

### Force and SEMG recording

**Fig. 1:**
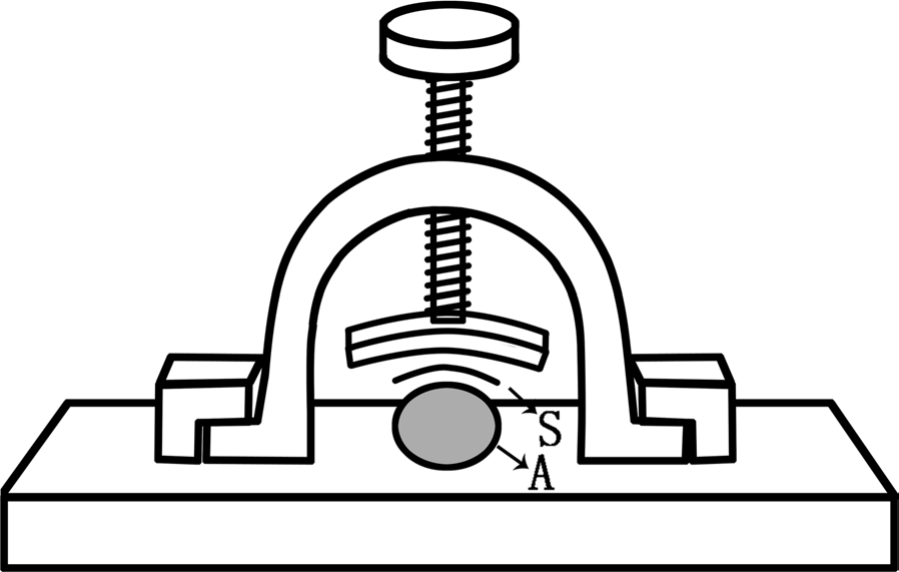
Diagrammatic sketch of a custom-made polymer plexiglass device designed for forearm -raising force detection. The base adhered to the table. The subject was required to sit in front of the table, fitting her left forearm onto the base. The arched superstructure was locked after a piece of T-Scan sensor (S) was placed over the forearm (A)

A T-Scan III System (Tekscan, Inc., Boston, MA, USA) was used to record the force sensitivity during clenching [19] and forearm raising (Fig. 1). Surface EMG activities from the bilateral TA and MM and the nondominant (left) side biceps brachii muscle (BicL) were simultaneously recorded using a BioEMG III electromyography recording system (Bioresearch Associates, Inc., Milwaukee, WI, USA) with a T-Scan system and T-Scan/BioEMG linking software (Tekscan, Inc., Bioresearch Associates technology partnership). For BicL, the electrode was positioned on the upper forearm's anterior (volar) surface, approximately 9 cm proximal to the elbow flexor fold [20]. According to generally accepted standards [19], surface electrodes (BioFLEX; Bioresearch Associates, Inc., Milwaukee, WI, USA) with 2 conductive polyester adhesive rectangular contacts of 144 square millimetres (mm^2^), each with a 20 mm centre-to-centre spacing, were placed on the skin after cleaning with 95% alcohol in the centre of each muscle area according to the BioPAK User Guide (ver. 7.0-2011). A common electrode (38 mm × 32 mm; Bioresearch Associates, Inc., Milwaukee, WI, USA) was placed on the back of the neck as a reference [21]. The SEMG signals were amplified differentially with a fixed gain of 5000, sampled at 1000 per second, and filtered in the bandwidth 20 Hz-1 kHz, as detailed in a recent publication [21]. The SEMG values representing target force levels corresponding to each task were stored for statistical analysis.

### Tasks

For clenching tasks, each subject was instructed to perform a fast clench from the resting position to ICP following three hypothetical force levels: mild as if biting a cake for Group I tasks (Task #1 to #4), medium as if biting peanuts for Group II tasks (Task #5–#8), and maximum voluntary clenching (MVC) for Group III tasks (Task #9–#12). The force values in each of the three groups were required to remain at the level of Tasks #1, #5, and #9 and to be as close as possible. Tasks #1, #5, and #9 were performed without visual feedback or maintenance. The highest force scale values of each of these three tasks displayed on the screen were 395.4 ± 489.6, 1611.9 ± 1160.7, and 5219.1 ± 4440.3, respectively. It is necessary to refer to this set of force values to achieve similar force values and so-called visual feedback. Tasks #2, #6, and #10 were performed with visual feedback but without any maintenance effort. Tasks #3, #7, and #11 were performed with maintenance effort for 5 s but without visual feedback. Tasks #4, #8, and #12 were performed with maintenance effort for 5 s via visual feedback.

Similarly, 12 forearm raising tasks were performed from rest vertically to the hypothetical low (as if lifting a basketball), medium (as if lifting a watermelon), and maximum levels with or without visual feedback and/or maintenance effort. The highest recorded values of the hypothetical low, medium, and high force levels were 91.44 ± 67.3, 626.76 ± 741.9, and 2337.6 ± 1921.5, respectively. Again, referring to this set of force values is necessary to achieve similar force values through visual feedback.

Different pieces of the T-Scan sensor were used for forearm raising and clenching, 3 to 4 pieces per person. Tasks were performed first for forearm raising and clenching, starting from light to maximum level. To avoid fatigue, a 5 min interval for rest was taken between every two tasks. The valid recordings evaluated by the examiner (LJ) were saved for further analysis.

### Evaluation of the degree of the uptight responses and task difficulty

Two questions required responses immediately after each task: 1) “How do you rate the difficulty of accomplishing this task?” and 2) “How do you rate the uptight response of accomplishing this task?” The subjects' experiences were rated on a 10 cm visual analogue scale (VAS) with a lower limit of "no difficulty" or "no tension response" and an upper limit of "most difficult" or "most tension response imaginable". The VAS values were used for further statistical analysis (See Additional file 1 for details).

### Statistical analysis

The SPSS 18.0 package (SPSS Co., Chicago, IL. USA) was used to describe and analyse the data. A linear regression analysis was performed to investigate the relationship between difficulty and uptight responses. We used the factorial design instead of the repeated measurement design for this trial. Factorial design ANOVA was performed for comparisons of VAS scores or SEMG activity with the associations of the following three factors, and the partial eta squared (η_p_^2^) was used to examine the within-group effect sizes: Factor 1, force levels: low, medium, and maximum; Factor 2, visual feedback: with and without; Factor 3, force maintenance: with and without. The factor interaction was also assessed. Post hoc tests were performed using Tukey’s test for intragroup comparison when a difference was found. The significance level was set at *P* < 0.05 for all statistical tests.

## Results

### Task-induced uptight responses are associated with task difficulty

**Fig. 2:**
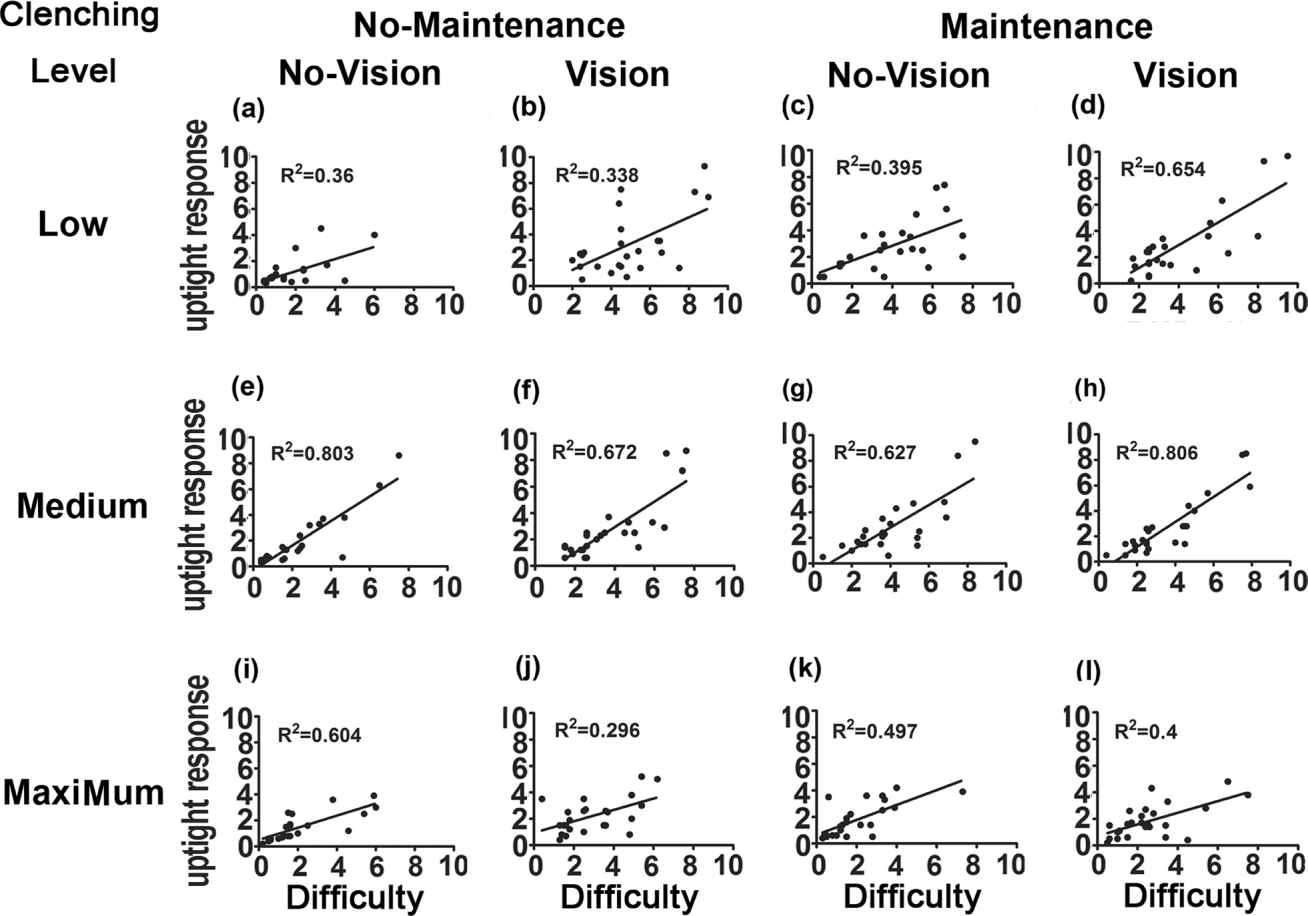
Correlation of the task difficulty and uptight response levels elicited during clenching at low (**a**, **b**, **c**, **d**), medium (**e**, **f**, **g**, **h**), and maximum force levels (**i**, **j**, **k**, **l**) with or without visual feedback and with or without maintenance effort. No maintenance = without maintenance effort; Maintenance   = with maintenance effort; No vision = without visual feedback; vision =  with visual feedback. R^2^ = coefficient of determination

**Fig. 3:**
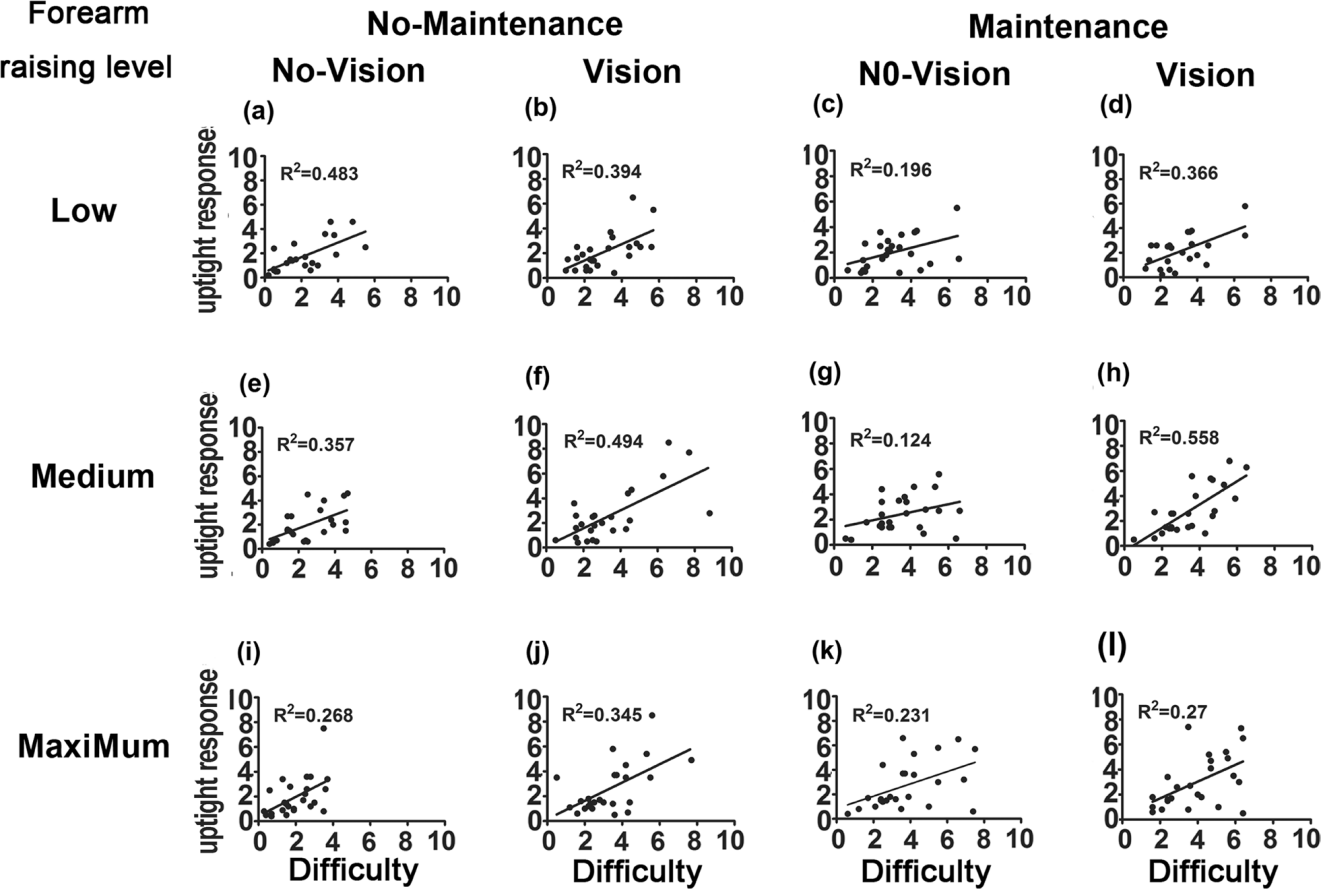
Correlation of the task difficulty and uptight response levels elicited during forearm raising at low (**a**, **b**, **c**, **d**), medium (**e**, **f**, **g**, **h**), and maximum force levels (**i**, **j**, **k**, **l**) with or without visual feedback and with or without maintenance effort. No maintenance  =  without maintenance effort; Maintenance = with maintenance effort; No vision = without visual feedback; vision = with visual feedback. R^2^  = coefficient of determination

The uptight response VAS value was generally correlated with the difficulty VAS value (*P* < 0.05), the only exception being the forearm raising task at a medium force level, with maintenance but no visual feedback (P = 0.084, Figs. 2 and 3) (See Additional file 2 for details).

### Force level, visual feedback, and maintenance effort showed different effects on the VAS value of uptight responses

#### Clenching tasks

**Fig. 4:**
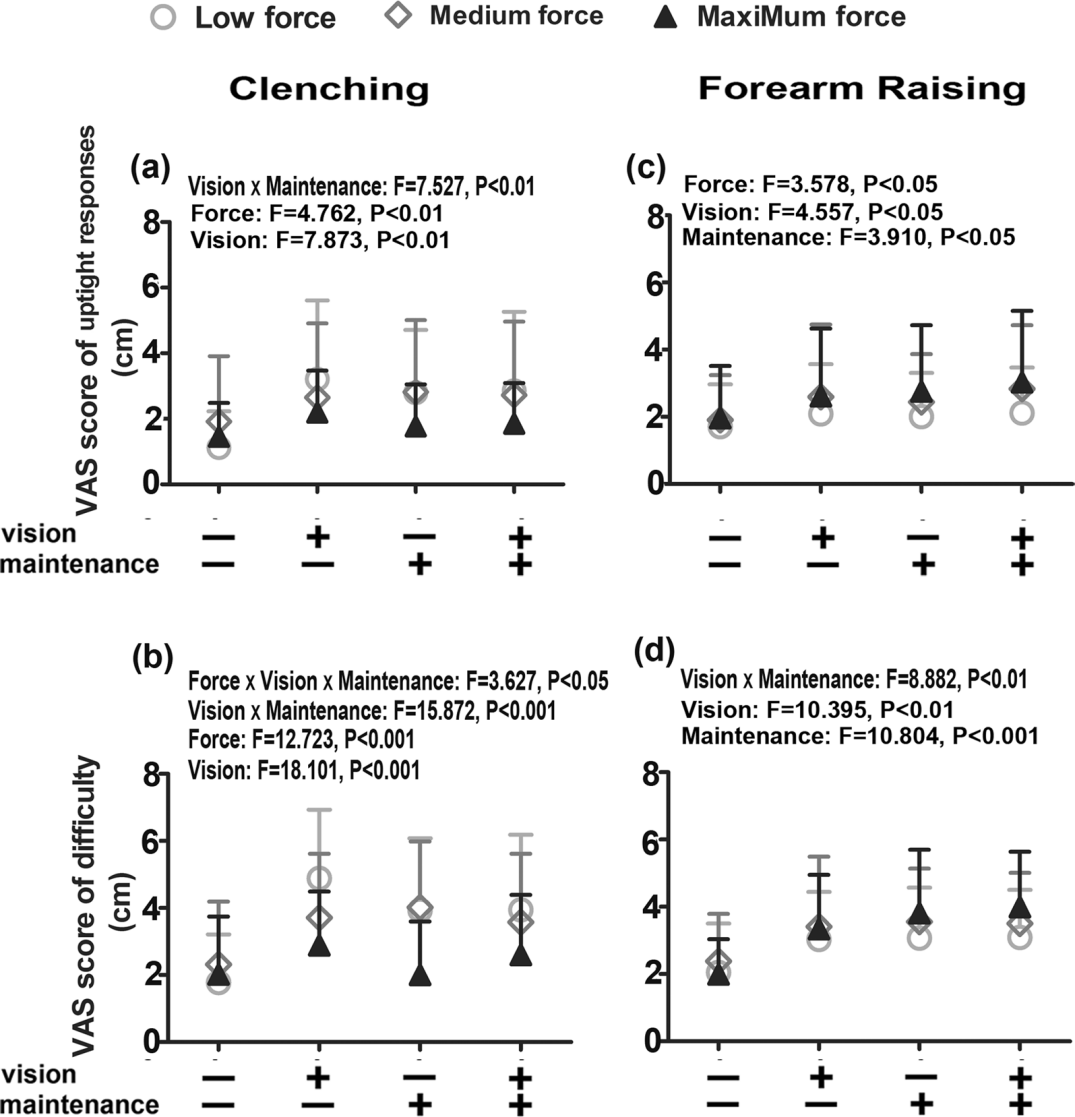
Means ± SDs of the uptight response visual analogue scale (VAS) scores (**a**, **c**) and the difficulty VAS scores (**b**, **d**) during clenching (**a**, **b**) and forearm raising (**c**, **d**) at low, medium, and maximum force levels with or without visual feedback and with or without maintenance effort. The multivariate factorial variance analysis results are presented in the panels

Factorial design ANOVA indicated that the force level and visual feedback factors contributed to the clenching uptight response VAS values (Fig. 4a, *P* < 0.01) and clenching difficulty VAS values (Fig. 4b, *P* < 0.001). In addition, visual feedback and maintenance effort showed an interaction effect on the uptight response VAS value (Fig. 4a, *P* = 0.006) and the difficulty VAS value (Fig. 4b*, P*< 0.001). An interaction of force level, visual feedback, and maintenance effort on difficulty values was also observed (Fig. 4b, *P* = 0.028). The effect sizes (i.e., ηp^2^) are listed in Table 1. Post hoc tests showed a negative link between the uptight response VAS value and force level. The highest VAS values of uptight responses and clenching difficulty were obtained when clenching occurred at the low force level with visual feedback but without maintenance effort. The dependent's partial eta square (ηp^2^) was low in many significant groups. Nevertheless, it showed that the force factor played the most crucial role in both clenching uptight response VAS values and difficulty VAS values, followed by visual feedback. The interaction between visual feedback and maintenance effort had the weakest effect.

**Table 1:** Outcomes from multivariate factorial variance analysis for the effects of force level, visual feedback, and maintenance effort on uptight responses and difficult VAS values and the surface electromyographic (SEMG) activity values of the bilateral anterior temporalis (TA) and masseter (MM) muscles during clenching and the SEMG activity values of the left biceps brachii muscle (BicL) during forearm raising

		Force	Visual feedback	Maintenance effort	Force level X Visual feedback	Force level X Maintenance effort	Visual feedback X Maintenance effort	Force level X Visual feedback X Maintenance effort
Clenching uptight responses	F	4.762	7.873	3.180	1.253	0.898	7.527	1.021
	*p*	0.009^**^	0.005^**^	0.076	0.287	0.409	0.006^**^	0.362
	η_p_^2^	0.032	0.027	0.011	0.009	0.006	0.025	0.007
Forearm raising uptight responses	F	3.578	4.557	3.910	0.213	0.429	0.652	0.008
	*p*	0.029^*^	0.034^*^	0.049^*^	0.809	0.652	0.420	0.992
	η_p_^2^	0.024	0.016	0.013	0.001	0.003	0.002	0.000
Clenching difficulty	F	12.723	18.101	3.633	2.245	1.759	15.872	3.627
	*p*	0.000^***^	0.000^***^	0.058	0.108	0.174	0.000^***^	0.028^*^
	η_p_^2^	0.081	0.059	0.012	0.015	0.012	0.052	0.025
Forearm raising difficulty	F	2.701	10.395	19.804	0.261	1.447	8.882	.030
	*p*	0.069	0.001^**^	0.000^***^	0.771	0.237	0.003^**^	0.971
	η_p_^2^	0.018	0.035	0.064	0.002	0.010	0.030	0.000
TAR SEMG	F	93.913	3.033	32.103	0.713	8.473	1.083	0.745
	*p*	0.000^***^	0.083	0.000^***^	0.491	0.000^***^	0.299	0.476
	η_p_^2^	0.395	0.010	0.100	0.005	0.056	0.004	0.005
TAL SEMG	F	116.217	4.502	35.922	0.448	11.857	0.390	0.143
	*p*	0.000^***^	0.035^*^	0.000^***^	0.639	0.000^***^	0.533	0.867
	η_p_^2^	0.447	0.015	0.111	0.003	0.076	0.001	0.001
MMR SEMG	F	95.124	4.384	23.561	0.570	9.672	0.035	0.292
	*p*	0.000^***^	0.037^*^	0.000^***^	0.566	0.000^***^	0.851	0.747
	η_p_^2^	0.398	0.015	0.076	0.004	0.063	0.000	0.002
MML SEMG	F	92.371	2.575	21.296	0.718	7.511	0.706	0.102
	*p*	0.000^***^	0.110	0.000^***^	0.488	0.001^**^	0.401	0.903
	η_p_^2^	0.391	0.009	0.069	0.005	0.050	0.002	0.001
BicL SEMG	F	20.870	0.088	0.350	0.195	0.241	0.314	0.178
	*p*	0.000^***^	0.766	0.555	0.823	0.786	0.576	0.837
	η_p_^2^	0.127	0.000	0.001	0.001	0.002	0.001	0.001

*p < 0.05, **p < 0.01,***p < 0.001

#### Forearm-raising tasks

Factorial design ANOVA indicated that force level, visual feedback, and maintenance effort raised uptight response VAS values (Fig. 4c,  *P* < 0.05) without any interactions. The factor of visual feedback and maintenance effort contributed to the forearm-raising difficulty values (Fig. 4d , *P*< 0.01) with an interaction between them (Fig. 4d, *P* = 0.003). The effect sizes (i.e., ηp^2^) are listed in Table 1. Post hoc tests showed that the highest VAS values of forearm-raising uptight response and difficulty were obtained when the task was performed at the maximum force level with visual feedback and maintenance effort. The dependent's partial eta square (ηp2) was low in many significant groups but provided order information on their effect intensity. Based on the information provided, for the forearm raising uptight response VAS values, the effect intensity showed a decreasing order: force, visual feedback, and maintenance. However, forearm-raising difficulty values were maintenance, visual feedback, and interaction of visual feedback and maintenance.

### SEMG values increased with force level and were enhanced with maintenance effort

**Fig. 5:**
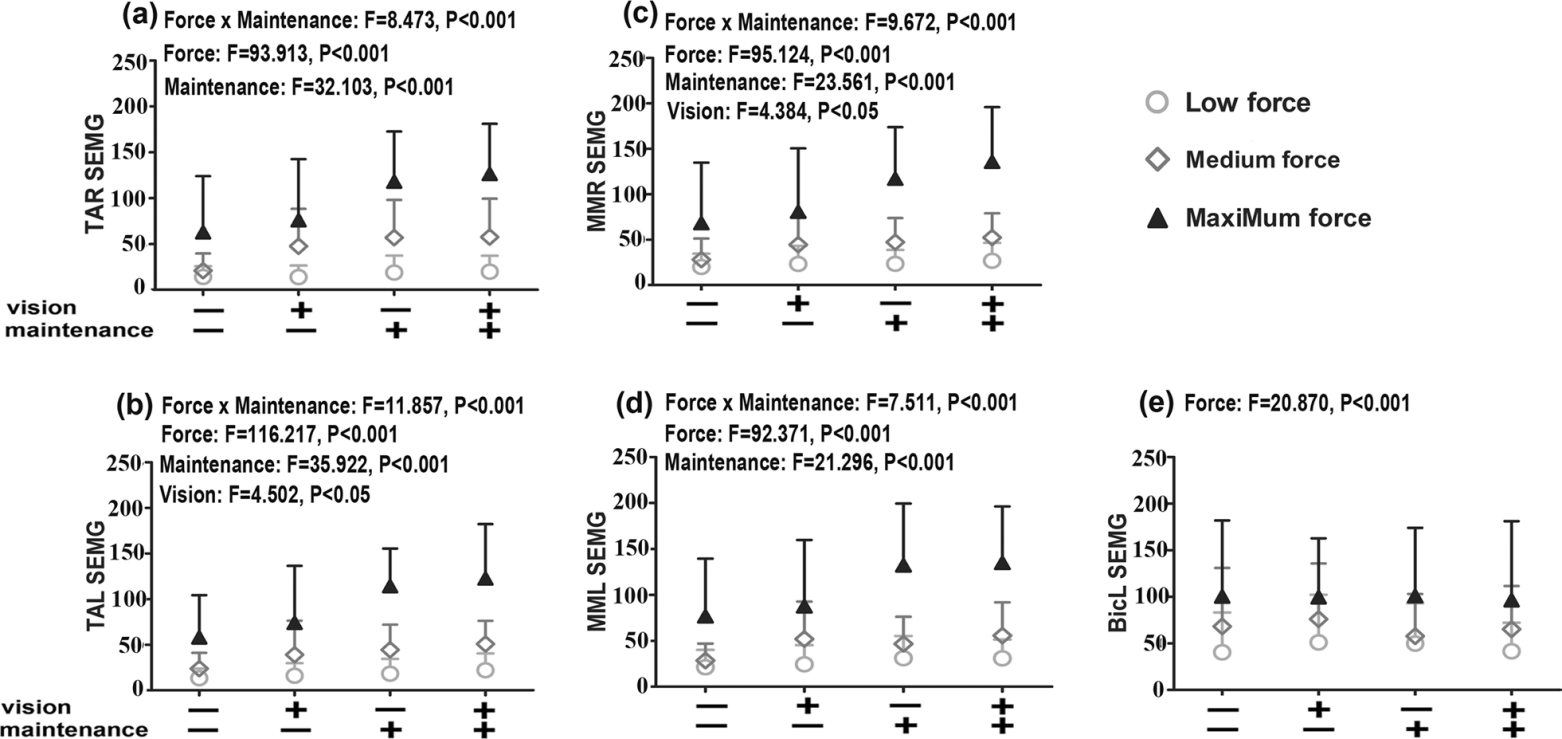
Mean ± SD of the SEMG activity values of the bilateral anterior temporalis (TA) and masseter (MM) muscles (**a**, **b**, **c**, **d**) during clenching in the intercuspal position (ICP) and of the left biceps brachii (BicL) muscle (**e**) during forearm raising at low, medium, and maximum force levels with or without visual feedback and with or without maintenance effort. The multivariate factorial variance analysis results are presented in the panels

Factorial design ANOVA indicated that the factor of force level and maintenance effort contributed, with an interaction (*P* < 0.01), to the SEMG values of the jaw muscles during clenching (*P* < 0.001, Fig. 5a–d). There was also a contribution from the factor of visual feedback to the SEMG values in TAL and MMR (*P* < 0.05, Fig. 5b, c). The effect sizes (i.e., ηp^2^) are listed in Table 1. The dependent's partial eta square (ηp^2^) showed that the effect intensity in decreasing order was force, maintenance, and interaction of force and maintenance for SEMG values in TAR and MML. For forearm-raising difficulty values, it was the force, maintenance, interaction of force and maintenance, and visual feedback (See Additional file 3 for details).

Post hoc tests showed that the values of the jaw muscle SEMG activity during clenching at the maximum level were consistently higher than those at the low force level (all *P* < 0.01) and were also higher than those at the medium force level under conditions without visual feedback and maintenance effort (*P* < 0.05), with or without visual feedback but with maintenance effort (*P* < 0.001), and with visual feedback and without maintenance effort (for TAL only, *P* = 0.029). The values at the medium force level did not display differences from those at a low force level (*P* > 0.05). The factor of maintenance enhanced the SEMG activity at the maximum force level with or without visual feedback (*P* < 0.01) but not at the medium or low force levels (all *P* > 0.05).

For BicL, the force-level factor contributed to the increase in SEMG activity (Fig. 5e, *P* < 0.001), but visual feedback and maintenance did not (*P* > 0.05). No interaction was observed (*P* > 0.05). The effect sizes (i.e., ηp^2^) are listed in Table 1. Post hoc tests showed higher SEMG activity at the maximum force level than at the low or medium force level (all *P* < 0.001), and the activity of SEMG at the moderate force level was higher than that at the low force level (*P* = 0.033).

## Discussion

The current data revealed that during either clenching or forearm raising, a high level of uptight response was elicited when the task difficulty degree was high. The highest VAS values of clenching difficulty were achieved when clenching occurred at the low force level with visual feedback but without maintenance effort. In contrast, the highest VAS value of forearm-raising difficulty was achieved when that task was performed at the maximum force level with visual feedback and maintenance effort. Consequently, the clenching-induced uptight responses showed a different regularity from forearm raising regarding the effect of force level, visual feedback, and maintenance. During forearm raising, a high-level uptight response was elicited when a high force level was maintained via visual feedback. However, a high uptight response was elicited when a low force level was targeted via visual feedback without maintenance during clenching. Maintenance effort affected forearm-raising-elicited uptight responses when functions as a single factor generally had no significant effect on clenching-elicited uptight responses.

Voluntary clenching and forearm raising are achieved by the contraction of jaw-closing muscles and limb muscles, respectively, and there is peripheral feedback regulation. When limb muscles contract, Golgi tendon organs will elicit negative feedback. However, few Golgi organs exist in jaw-closing muscles [22, 23]. Periodontal mechanoreceptors distributed throughout the roots assume the role of continuous feedback modification in the jaw-closing muscles [24]. Furthermore, the jaw-closing muscles are anatomically and histologically different from limb muscles. Most jaw-closing muscles are multipennate, complexly layered, and interleaved by aponeuroses. In contrast, limb muscles (for example, the fusiform biceps brachii muscles) have most muscle fibres parallel to the longitudinal muscle axis [22, 23].

Furthermore, most motor unit territories of the jaw muscles are smaller than those in the limbs [25]. In skeletal muscle, the fusiform receptors composed of intrafusal muscle fibres are called muscle spindles, and these spindles can sense the state of the muscle, maintain muscle tension, and finely regulate muscle movements in multiple directions so that the length, speed, and speed changes of the extrafusal muscle fibres can be adjusted at rest or during movement. The distribution of muscle spindles in skeletal muscles is uneven, and the difference in distribution density may be related to the different regulations of muscle physiological function. Skeletal muscles with high muscle spindle density are better at fine regulation, while muscles with low muscle spindle density tend to exercise with a large amplitude. It is believed that short and small muscles contain higher muscle spindle density than large muscles [26]. The maxillary muscle is more sensitive to peripheral feedback regulation to adapt to the complexity of various functional movements. It can quickly make avoidance muscle movements and adjust occlusal force according to adverse feedback information, which benefits the maintenance of masticatory system function. The key to maintaining the normal function of the stomatognathic system is to form the minimal damage mode of the subconscious chewing habit, which quickly receives input information from the central nervous system and peripheral feedback. All these characteristics imply a functional requirement of subtle modification of the jaw-closing muscles compared with the limb muscles—a process that involves periodontal feedback mechanisms. Further subtle regulation is required when the visual feedback-modified target low force level is aimed, leading to high-level difficulty, as indicated by the present data. This means that although the complete mashing and chewing of foods will cause delight, poor occlusion (which prevents direct and forceful chewing) would be more likely to elicit uptight responses.

Interestingly, maintenance effort, as a single factor, did not increase the uptight response level during clenching but increased the jaw-muscle SEMG activity when clenched with the maximum force effort. This implies that the bruxism or grinding habit, which is associated with high jaw-muscle activity, occurs mainly in the subconscious state and plays a minor role in uptight responses unless it works together with peripheral feedback regulation, given that the maintenance effort displayed an interaction effect with visual feedback. Abnormal occlusion, such as occlusal interference, may promote mental and psychological changes, and the SEMG activity level of the maxillary muscle may not be significantly increased. The central nervous system and peripheral feedback afferent regulation of the jaw muscle are complex and fine [27].

The change in psychological states may also be facilitated by a marked increase in maxillary muscle activity at the force level that can be better controlled through visual feedback. If the occlusal contact is poor, the periodontal feedback mechanism may need additional regulatory input to coordinate the mandibular movement in the most appropriate masticatory activity mode. These efforts make normal occlusal movement more difficult or lead to an uptight response or psychological changes. However, the visual feedback factor was a weak factor affecting the SEMG activity of the maxillary muscle in this experiment.

There are significant individual differences in the body's perception of stressors, and there are many release pathways. In the natural occlusal state, there is enough time for EMG to adjust and produce an adaptive occlusion [5], but when the occlusion cannot achieve a close and stable state through visual system regulation, occlusal adaptation, and adaptive reconstruction of temporomandibular joint (TMJ) structures, it may increase abnormal mental and psychological changes. Whether the difficulty in chewing smoothly is the initial factor of psychological state change and further promotes the malaise of TMDs needs further research and demonstration. It is assumed that the gap in muscle characteristics between men and women is relatively large. Clinically, occlusal motor discomfort is more common in women [17]. No male subjects were recruited for this study, which is indeed a limitation that needs to be explored in future studies.

## Conclusions

Conclusively, the present data indicate that different from the forearm-raising task, a higher uptight response is associated with a higher force level maintained via visual feedback. A higher uptight response to clenching is associated with a preset lower force level with visual feedback modification. This is clinically significant because patients with interferential occlusion that prevents forceful biting may suffer from psychological responses such as an uptight response.

## Supplementary Information

**Additional file 1**. Visual Analogue Scale.

**Additional file 2**. Raw data of  VAS.

**Additional file 3**. Raw data of SEMG.

## Data Availability

The study data are available from the corresponding author upon reasonable request.

## Abbreviations

SEMG: Surface electromyography
TA: Anterior temporalis
TAL: Left anterior temporalis
TAR: Right anterior temporalis
MM: Masseter muscle
MML: Left masseter muscle
MMR: Right masseter muscle
BicL: Left biceps brachii muscle
TMDs: Temporomandibular disorders
OHRQoL: Oral health-related quality of life
VAS: Visual analogue scale
MVC: Maximum voluntary clenching
ICP: Intercuspal position
TMJ: Temporomandibular joint
