# Supplementary material for: Uptight responses between clenching and forearm raising with factors of visual feedback and maintenance effort in healthy young women: An experimental study on factorial design
Source: BMC Oral Health. 2023 Feb 14;23:98. doi: 10.1186/s12903-023-02767-9 (PMC9926863; doi:10.1186/s12903-023-02767-9)
Supplement: Supplementary file 1 — Additional file 1. Visual Analogue Scale. [file 12903_2023_2767_MOESM1_ESM.doc]

Name

Force (Low Medium Maximum )

1、clench no visual feedback no maintenance effort

How do you rate the difficulty of accomplishing this task?

0 1 2 3 4 5 6 7 8 9 10

|  |  |  |  |  |  |  |  |  |  |
| --- | --- | --- | --- | --- | --- | --- | --- | --- | --- |

How do you rate the uptight response of accomplishing this task?

0 1 2 3 4 5 6 7 8 9 10

|  |  |  |  |  |  |  |  |  |  |
| --- | --- | --- | --- | --- | --- | --- | --- | --- | --- |

2、clench visual feedback no maintenance effort

How do you rate the difficulty of accomplishing this task?

0 1 2 3 4 5 6 7 8 9 10

|  |  |  |  |  |  |  |  |  |  |
| --- | --- | --- | --- | --- | --- | --- | --- | --- | --- |

How do you rate the uptight response of accomplishing this task?

0 1 2 3 4 5 6 7 8 9 10

|  |  |  |  |  |  |  |  |  |  |
| --- | --- | --- | --- | --- | --- | --- | --- | --- | --- |

3、 clench no visual feedback maintenance effort

How do you rate the difficulty of accomplishing this task?

0 1 2 3 4 5 6 7 8 9 10

|  |  |  |  |  |  |  |  |  |  |
| --- | --- | --- | --- | --- | --- | --- | --- | --- | --- |

How do you rate the uptight response of accomplishing this task?

0 1 2 3 4 5 6 7 8 9 10

|  |  |  |  |  |  |  |  |  |  |
| --- | --- | --- | --- | --- | --- | --- | --- | --- | --- |

4、clench visual feedback maintenance effort

How do you rate the difficulty of accomplishing this task?

0 1 2 3 4 5 6 7 8 9 10

|  |  |  |  |  |  |  |  |  |  |
| --- | --- | --- | --- | --- | --- | --- | --- | --- | --- |

How do you rate the uptight response of accomplishing this task?

0 1 2 3 4 5 6 7 8 9 10

|  |  |  |  |  |  |  |  |  |  |
| --- | --- | --- | --- | --- | --- | --- | --- | --- | --- |

5、forearm raise no visual feedback no maintenance effort

How do you rate the difficulty of accomplishing this task?

0 1 2 3 4 5 6 7 8 9 10

|  |  |  |  |  |  |  |  |  |  |
| --- | --- | --- | --- | --- | --- | --- | --- | --- | --- |

How do you rate the uptight response of accomplishing this task?

0 1 2 3 4 5 6 7 8 9 10

|  |  |  |  |  |  |  |  |  |  |
| --- | --- | --- | --- | --- | --- | --- | --- | --- | --- |

6、forearm raise visual feedback no maintenance effort

How do you rate the difficulty of accomplishing this task?

0 1 2 3 4 5 6 7 8 9 10

|  |  |  |  |  |  |  |  |  |  |
| --- | --- | --- | --- | --- | --- | --- | --- | --- | --- |

How do you rate the uptight response of accomplishing this task?

0 1 2 3 4 5 6 7 8 9 10

|  |  |  |  |  |  |  |  |  |  |
| --- | --- | --- | --- | --- | --- | --- | --- | --- | --- |

7、forearm raise no visual feedback maintenance effort

How do you rate the difficulty of accomplishing this task?

0 1 2 3 4 5 6 7 8 9 10

|  |  |  |  |  |  |  |  |  |  |
| --- | --- | --- | --- | --- | --- | --- | --- | --- | --- |

How do you rate the uptight response of accomplishing this task?

0 1 2 3 4 5 6 7 8 9 10

|  |  |  |  |  |  |  |  |  |  |
| --- | --- | --- | --- | --- | --- | --- | --- | --- | --- |

8、forearm raise visual feedback maintenance effort

How do you rate the difficulty of accomplishing this task?

0 1 2 3 4 5 6 7 8 9 10

|  |  |  |  |  |  |  |  |  |  |
| --- | --- | --- | --- | --- | --- | --- | --- | --- | --- |

How do you rate the uptight response of accomplishing this task?

0 1 2 3 4 5 6 7 8 9 10

|  |  |  |  |  |  |  |  |  |  |
| --- | --- | --- | --- | --- | --- | --- | --- | --- | --- |
